# Temporal and Spatial Impact of Human Cadaver Decomposition on Soil Bacterial and Arthropod Community Structure and Function

**DOI:** 10.3389/fmicb.2017.02616

**Published:** 2018-01-04

**Authors:** Baneshwar Singh, Kevan J. Minick, Michael S. Strickland, Kyle G. Wickings, Tawni L. Crippen, Aaron M. Tarone, M. Eric Benbow, Ness Sufrin, Jeffery K. Tomberlin, Jennifer L. Pechal

**Affiliations:** ^1^Department of Forensic Sciences, Virginia Commonwealth University, Richmond, VA, United States; ^2^Department of Forestry and Environmental Resources, North Carolina State University, Raleigh, NC, United States; ^3^Department of Soil and Water Systems, University of Idaho, Moscow, ID, United States; ^4^Department of Entomology, Cornell University, Geneva, NY, United States; ^5^Southern Plains Agricultural Research Center, Agricultural Research Service, United States Department of Agriculture, College Station, TX, United States; ^6^Department of Entomology, Texas A&M University, College Station, TX, United States; ^7^Department of Entomology and Department of Osteopathic Medical Specialties, Michigan State University, East Lansing, MI, United States; ^8^Bode Cellmark Forensics, Lorton, VA, United States; ^9^Department of Entomology, Michigan State University, East Lansing, MI, United States

**Keywords:** decomposition ecology, soil microbiology, postmortem interval, soil arthropods, soil biodiversity, grave soil

## Abstract

As vertebrate carrion decomposes, there is a release of nutrient-rich fluids into the underlying soil, which can impact associated biological community structure and function. How these changes alter soil biogeochemical cycles is relatively unknown and may prove useful in the identification of carrion decomposition islands that have long lasting, focal ecological effects. This study investigated the spatial (0, 1, and 5 m) and temporal (3–732 days) dynamics of human cadaver decomposition on soil bacterial and arthropod community structure and microbial function. We observed strong evidence of a predictable response to cadaver decomposition that varies over space for soil bacterial and arthropod community structure, carbon (C) mineralization and microbial substrate utilization patterns. In the presence of a cadaver (i.e., 0 m samples), the relative abundance of Bacteroidetes and Firmicutes was greater, while the relative abundance of Acidobacteria, Chloroflexi, Gemmatimonadetes, and Verrucomicrobia was lower when compared to samples at 1 and 5 m. Micro-arthropods were more abundant (15 to 17-fold) in soils collected at 0 m compared to either 1 or 5 m, but overall, micro-arthropod community composition was unrelated to either bacterial community composition or function. Bacterial community structure and microbial function also exhibited temporal relationships, whereas arthropod community structure did not. Cumulative precipitation was more effective in predicting temporal variations in bacterial abundance and microbial activity than accumulated degree days. In the presence of the cadaver (i.e., 0 m samples), the relative abundance of Actinobacteria increased significantly with cumulative precipitation. Furthermore, soil bacterial communities and C mineralization were sensitive to the introduction of human cadavers as they diverged from baseline levels and did not recover completely in approximately 2 years. These data are valuable for understanding ecosystem function surrounding carrion decomposition islands and can be applicable to environmental bio-monitoring and forensic sciences.

## Introduction

Our understanding of the spatial and temporal extent of vertebrate carrion decomposition on soil communities and microbial ecology is still very limited. This is particularly true for human cadavers, which have rarely been studied in an ecosystem context. A few recent studies have investigated the effects of human (Cobaugh et al., 2015; Metcalf et al., 2016) and non-human vertebrate decomposition on underlying soils (Bump et al., 2009; Parmenter and MacMahon, 2009; Barton et al., 2016; Metcalf et al., 2016; Szelecz et al., 2016) in an ecological context, but a more comprehensive analysis of soil biota is still lacking (e.g., long term impact on microarthropods and soil microorganisms). Previous examinations of human cadaver decomposition have mostly emphasized direct applications to forensic sciences (Rodriguez and Bass, 1983; Megyesi et al., 2005). While critical, this narrow scope overlooks the ecosystem and community level impacts on soil biochemistry, nutrient cycling, and biological community structure (Carter et al., 2007). The world is facing several global challenges which may cause increased inputs into ecosystems, particularly with increased regional conflicts amongst human populations (Hsiang et al., 2011). Apart from human remains, the frequency of mass mortality events of other vertebrate species also appears to be on the rise (Fey et al., 2015), requiring a better understanding of the impacts of cadaver-derived, nutrient rich inputs on soil communities and other ecosystem processes (Vass et al., 1992; Parkinson et al., 2009).

The process of human cadaver decomposition itself has been widely studied, primarily as an indicator of time since death. Cadaver decomposition starts immediately after death with cellular autolysis progressing to putrefaction (Dent et al., 2004; Crippen and Singh, 2015) caused by microbes associated with the cadaver itself (Pechal et al., 2013), and altered by surrounding environmental components, such as the soil (Lauber et al., 2014; Pechal et al., 2014), insects (Zheng et al., 2013; Singh et al., 2014; Benbow et al., 2015), and vertebrate scavengers (DeVault and Rhodes, 2002; Roggenbuck et al., 2014). As decomposition proceeds, anaerobic bacteria primarily within the intestinal tract, produce gases resulting in bloating followed by the purge of fluids and subsequent release of nutrients and microbes into the underlying soil (Metcalf et al., 2013). Introduction of cadaver-associated microorganisms to underlying soils may also alter soil microbial community composition (Cobaugh et al., 2015). The use of model systems [i.e., pig (Benninger et al., 2008) and mice (Metcalf et al., 2013)] across short time scales (i.e., 40–100 days) has already demonstrated marked changes in soil chemical, physical, and biological properties; especially an increases of phosphorus, sodium, nitrogen, and pH (Melis et al., 2007; Parmenter and MacMahon, 2009). Such changes, influenced by local climate factors (Carter et al., 2007), likely have marked effects on soil function and the composition of invertebrate and microbial communities. Unfortunately, studies on the impact of carrion decomposition on soil biological community structure and function is very limited (Parkinson et al., 2009).

The impact of carrion decomposition on arthropod and microbial community structure and function may go beyond the actual carcass boundaries (Benbow et al., 2015; Pechal and Benbow, 2015). For instance, we know that carrion inputs alter invertebrate and vertebrate scavenger community dynamics (Hocking et al., 2009; Cortes-Avizanda et al., 2012; Pechal and Benbow, 2016; Szelecz et al., 2016), and influence contiguous plant community composition, soil microbial biomass and soil chemistry (Yang, 2004). Such impacts are not necessarily ephemeral and may have long-term effects on ecosystem processes (Strickland and Wickings, 2015; Barton et al., 2016). Hawlena et al. (2012) demonstrated that after grasshopper carrion is completely decomposed, the legacy of this input subsequently influenced leaf litter decomposition at that location. Although it is very clear that the size of the decomposing organism influences the size of the cadaver decomposition island (CDI) (Coe, 1978; Barton et al., 2016), and cadaver mass has little influence on soil microbial community across short time scales (<15 days) (Weiss et al., 2016), it is not clear if the principles hold true for large carcasses (e.g., human remains) and across longer time scales (>1 year). These overlooked “multiplier effects” on communities and ecosystems (Schmitz et al., 2013) are likely to increase with the mortality of animals of larger mass or as a result of mass mortality events.

In this study, we assessed the effects of human cadaver decomposition on soil bacterial and arthropod communities by providing a more comprehensive analysis of soil biota and carbon (C) cycling than previously performed, and its potential to influence long-term (2 years) ecosystem function. We expected the underlying soil chemistry and biotic communities would closely follow the progression of cadaver decomposition and the influence would be most noticeable directly beneath the body and decrease with distance from the body, akin to carrion decomposition islands (Carter et al., 2007). Also we expected that during the initial stage of decomposition effects on the underlying soil would be minimal, then increase after purging, and slowly decrease to pre-cadaver soil conditions at some future point; the spatial and temporal dynamics to be dictated by abiotic (e.g., temperature and moisture) as well as biotic (e.g., body mass, indigenous microbes) factors. This information advances the understanding of the temporal and spatial effects of human cadaver decomposition on soil communities and processes, and has direct application to forensic science.

## Materials and Methods

### Site Description and Sample Collection

Eleven donated human cadavers (received under The Universal Anatomical Gift Act) were placed at the Forensic Anthropology Research Facility (FARF) of Texas State University, San Marcos, TX, United States (**Supplementary Table S1**). Institutional Review Board (IRB) approval was not required for use of human cadavers because no personal or identifiable information was collected. The FARF is a 26-acre outdoor human decomposition research laboratory located at Texas State’s Freeman Ranch, Hays County, TX, United States (29°55′56.2″ N, 97°59′57.3″ W) that lies within the Edwards Plateau physiographic region. This site is classified as a grassland/savanna ecosystem, which is characterized by grasslands and tree clusters of predominately live oak (*Quercus virginiana*) with some Ashe juniper (*Juniperus ashei*) trees (Baccus et al., 2000). Soils at the study site are classified as Comfort series (clayey-skeletal, mixed, superactive, thermic Lithic Argiustolls) and Rumple series (clayey-skeletal, mixed, active, thermic Typic Argiustolls) with underlying material of erosion-resistant limestone and limestone interbedded with clay and marl (Carson, 2000). Cadavers were placed with a minimum of 15 m between two bodies outside on mineral soils located on bare ground between patches of trees to decompose directly under natural conditions. All cadavers were placed in an area of approximately 23,000 m^2^. To minimize scavenging activity by vertebrate scavengers, a cage (approximately 5 cm × 5 cm mesh size) was placed on each cadaver.

Soil samples (0–10 cm deep) were collected from directly under cadavers using a 10 cm depth stainless steel soil corer (Forestry Suppliers, United States). Given the difficulties in obtaining human cadavers for research and in order to obtain the largest data set possible, human cadavers were placed out in the field at different dates between 2010 and 2012 and sampled once (seven cadavers) or three times (four cadavers) throughout 2012. This design allowed for soil samples to be collected at different time points after cadaver placement in the field ranging from 3 to 732 days (**Supplementary Table S1**). At each sampling date, three soil cores were collected from under cadavers (0 m) and composited, resulting in one soil sample per cadaver sampled and sampling date. Triplicate samples were also taken at two distances from each cadaver (1 and 5 m) and composited at each distance into a single Ziploc^®^ bag (S. C. Johnson & Son, Inc., United States), each weighing 0.9 to 1.4 kg, for a total of 19 samples collected at each of the three sampling locations (i.e., total of 57 samples were collected from all sampling locations). Between samples, the soil corer was cleaned with bottled water, then disinfected with Lysol^®^ wipes, and finally with DNA AWAY^TM^ wipes (Thermo Fisher Scientific, Inc., United States). Samples were transported to the lab in cold storage boxes containing ice packs. Subsamples were stored at -20°C for DNA extraction and at 4°C for microbial functional analysis and arthropod extraction.

Air temperature (°C) and precipitation (mm) data were downloaded from the National Oceanic and Atmospheric Administration (NOAA) climatic data records for San Marco, TX, United States (GHCND:USC00417983; 29°52′59.5194″ N, 97°56′57.8394″ W). Using these data, accumulated degree days (ADDs) and cumulative precipitation (mm) were calculated. ADD was calculated by summing daily average temperatures (°C) that measured over 0°C for each cadaver between the time of placement in the field and each subsequent sampling date (Vass et al., 1992; Megyesi et al., 2005) (**Supplementary Table S1**). The minimum temperature threshold of 0°C was determined given the expectation that microbial activity would most likely occur at or above 0°C in this arid region (Pechal et al., 2013; Strickland et al., 2015). Cumulative precipitation was calculated by summing the total amount of precipitation (mm) each cadaver was exposed to between time of placement in the field and subsequent sampling dates (**Supplementary Table S1**). The precipitation data were used as a predictor of microbial and arthropod community structure and function, given that the study site is in a dry region and therefore moisture is likely a key factor influencing cadaver decomposition and soil processes.

### Bacterial Community Composition

DNA was extracted from soil samples using a modified cetyltrimethyl ammonium bromide (CTAB) extraction method as described in Zheng et al. (2013). Once extracted, DNA was cleaned using PowerClean^®^ DNA Clean-Up Kit (MO BIO Laboratories, Inc., United States) and the concentration and purity of cleaned DNA were quantified using a NanoDrop 2000 spectrophotometer (Thermo Fisher Scientific, Inc., United States). Bacterial community composition was assessed via amplification of the bacterial variable region V1–V3 of 16S rRNA gene using the universal bacterial primer pair 27F (5′- GAGTTTGATCNTGGCTCAG) and 519R (5′- GTNTTACNGCGGCKGCTG). Amplicons were subjected to 454-pyrosequencing by bacterial tag-encoded FLX-Titanium pyrosequencing (bTEFAP) method (Dowd et al., 2008) in the Genome Sequencer FLX System (Roche, Inc., United States). All FLX related procedures were performed following Genome Sequencer FLX System manufacturers instructions (Roche, Nutley, NJ, United States). Sequencing errors from all sequences were minimized using program PyroNoise (Quince et al., 2011) as implemented in Mothur v 1.32 (Schloss et al., 2009). Low quality regions of the sequences (total count of sequences = 127944) were trimmed using a sliding window (50 bp; Q35) option in Mothur v 1.32 (Schloss et al., 2009). Sequence with homopolymer length > 8 bp, and total length < 250 bp were removed from further analyses (Schloss et al., 2011). USEARCH was used for quality filtering and clustering reads into operational taxonomic units (OTUs) at 97% identity threshold, following the customized UPARSE pipeline described in Edgar (2013). Taxonomy was assigned to OTUs at 97% identity threshold via the RDP classifier, using the GreenGenes 13.8 reference database for bacteria/archaea (DeSantis et al., 2006; Wang et al., 2007). QIIME version 1.8.0 was used to generate rarefied OTU tables (rarefaction cutoff = 134 count/sample) and Chao 1 diversity estimates (Caporaso et al., 2010). All raw sequence files were submitted to the European Nucleotide Archive (ENA) database as a part of the study PRJEB16630 (accession # ERS1420653 to ERS1420705).

### Soil Parameters and Microbial Function

Soil pH was analyzed by mixing soil and distilled H_2_O in a 1:1 ratio by volume and allowing the mixture to sit for 10 min before measuring with a bench top pH meter. Substrate induced respiration (SIR), an indicator of the active microbial biomass, was measured according to Fierer et al. (2003). In order to quantify microbial functional response, catabolic response profiles (CRP) were measured on all soil samples using multiple C substrates (glucose, glycine, sucrose, oxalic acid, chitin, and cellulose), which are indicative of C compounds typically found in soils and in order to stimulate community level responses to the various compounds, as outlined in Degens and Harris (1997). Briefly, we amended 4 g dry weight equivalent soil (1 analytical rep per solution) with 8 mL solutions of the C substrates in solution (each solution was adjusted to pH 6 using NaOH or HCl prior to addition). After a 1 h pre-incubation with shaking, the soil slurries (i.e., soil and solution combinations) were incubated for 4 h at 20°C, except for chitin and cellulose which were incubated for 24 h. CRPs were then determined as the percent contribution of respiration attributed to each C substrate to the total respiration derived from all of the C substrates. Furthermore, 30 days C mineralization assays were carried out in order to quantify total mineralizable-C, an estimate of microbially available C (Strickland et al., 2010) and microbial function. For SIR, CRP, and C mineralization assays, respiration was determined on an infrared gas analyzer (IRGA, Model LI-7000; Li-Cor Biosciences, Lincoln, NE, United States) using a static incubation technique.

### Soil Arthropod Extraction

Soil arthropods were extracted from approximately 100 g of field-moist soil retrieved from the same locations used for microbial analyses (0, 1, and 5 m). Soil samples were extracted for a 5-day period on collapsible Berlese funnels (BioQuip, United States). Extractions began at approximately 22°C and temperature was increased daily to a final temperature of 50°C. All arthropods were preserved in 90% ethanol for identification, and identifications were made to the level of family where possible using keys from Triplehorn and Johnson (2005). Collembolan families were determined using Dindal (1990) and following the taxonomic structure outlined in the pictoral key to Collembolan families from France by Janssens and Lebeaux (http://www.collembola.org/key/fkfr.htm; last updated on 05/31/2017), and oribatid, prostigmatid, and mesostigmatid mites were identified using the keys obtained from the Summer Acarology Course, hosted at Ohio State University. Arthropod densities are reported as the number of individuals 100 g^-1^ of soil.

### Statistical Analysis

The effect of distance from cadaver on soil bacterial community composition, function, and arthropod community composition was tested via permutation-MANOVA (PERMANOVA) and visualized using principal coordinates analysis (PCoA). Bray–Curtis distances were calculated for bacterial (based on OTU table) and arthropod composition while Euclidian distances were calculated for function. Specifically, for the PERMANOVA (Anderson, 2001) (9,999 permutations), distance from the cadaver was the independent variable, and cadaver identity (see **Supplementary Table S1**) was treated as a random effect to account for repeated sampling of soil from a subset of cadavers across time. Pair-wise differences between distances were also determined via PERMANOVA. Homogeneity of dispersions from the centroids was also determined, allowing the assessment of variation in composition or function associated with a given distance. To determine which component of microbial community composition or function contributed to differences between distances, the percentage contribution of taxa for community composition or of substrate for function to dissimilarity between distances (i.e., 0, 1, or 5 m) was determined using the SIMPER test in Primer (Clarke and Gorley, 2006). Mantel tests were used to examine relationships between bacterial community composition, function, and arthropod community composition.

Linear mixed effects models were used to assess the effect of distance on bacterial diversity, total arthropod abundance, and soil characteristics (i.e., soil pH, active microbial biomass, and mineralizable-C). Specifically, for each of these models, distance from the cadaver was the independent variable, and cadaver identity was treated as a random effect to account for repeated sampling of soil from a subset of cadavers across time. Pair-wise differences between distances were determined via Tukey’s HSD (*P* < 0.05) for bacterial data, and via LS means for arthropod data (as shown in **Supplementary Table S2**).

For the 0 m samples (i.e., those taken directly beneath the cadaver), a combination of linear and non-linear regression was used to assess the relationships between principal components axes and factors associated with temporal (i.e., accumulated degree day, cumulative precipitation) and cadaver specific dynamics (i.e., cadaver mass). If significant (*P* < 0.05) relationships with an axis were noted, this relationship was further examined by assessing the components of either composition or function associated with that axis. Additionally, linear and non-linear regression was used to assess the relationship between soil characteristics and temporal dynamics. Regression and linear mixed-effects models, using tem nlme package version 3.1-131, were conducted in R version 3.2.3 (R Core Team, 2012) and bacterial community, arthropod community, and functional analyses were conducted in Primer (Clarke and Gorley, 2006). Arthropod data shown in **Supplementary Table S2** were analyzed using SAS v9.3 (SAS Institute, Cary, NC, United States).

## Results

### Spatial Dynamics

**FIGURE 1:**
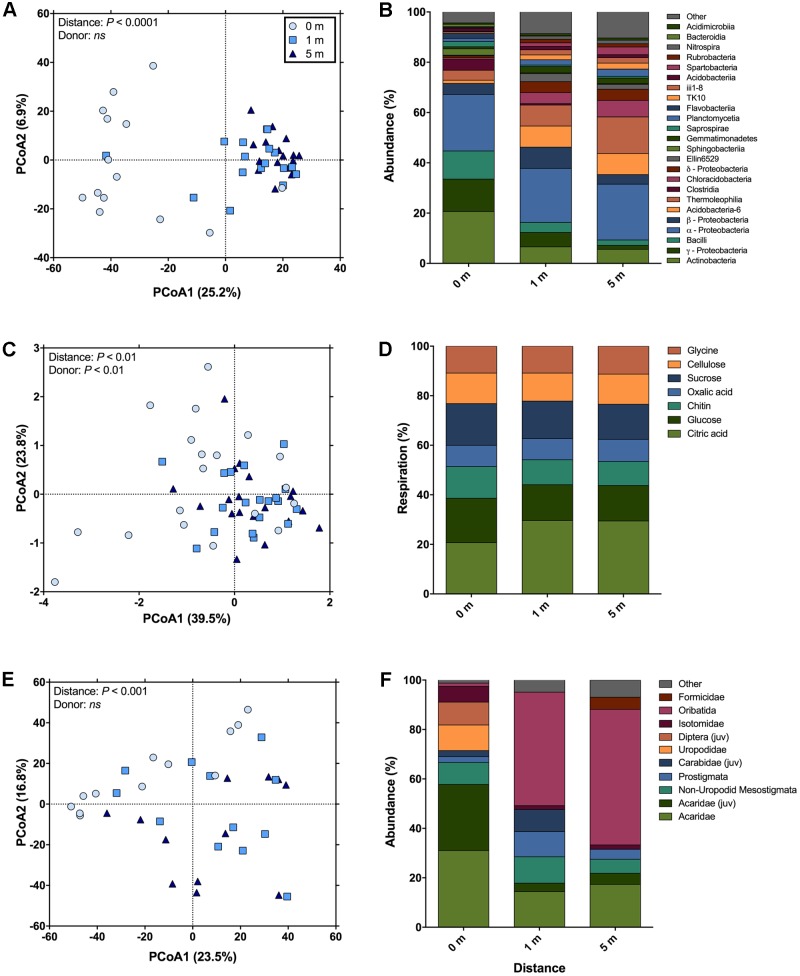
The change in soil community composition (bacteria and arthropod) and function with distance from human cadavers. **(A)** Bacterial community composition was significantly different at 0 m (i.e., under the cadaver) compared to 1 and 5 m from the cadaver. Bacterial communities at 5 m away from the cadaver were significantly less disperse than communities at 0 and 1 m. **(B)** Average compositional differences between bacterial communities at each measurement distance (*n* = 14 for 0 m distance and *n* = 17 for 1 and 5 m distances). **(C)** Catabolic response profiles (CRPs) at 0 m were significantly different from the 1 and 5 m distances. The 0 m communities were significantly more disperse than communities at 1 and 5 m. **(D)** Average differences in proportional respiration of C-substrates at each measurement distance (*n* = 19 for each distances). **(E)** Community composition of soil fauna was significantly different at 0 m compared to 1 and 5 m from the cadaver. No differences in dispersion between these distances were observed. **(F)** Average compositional differences between faunal communities at each measurement distance (*n* = 19 for each distances; “juv” means juvenile).

**FIGURE 2:**
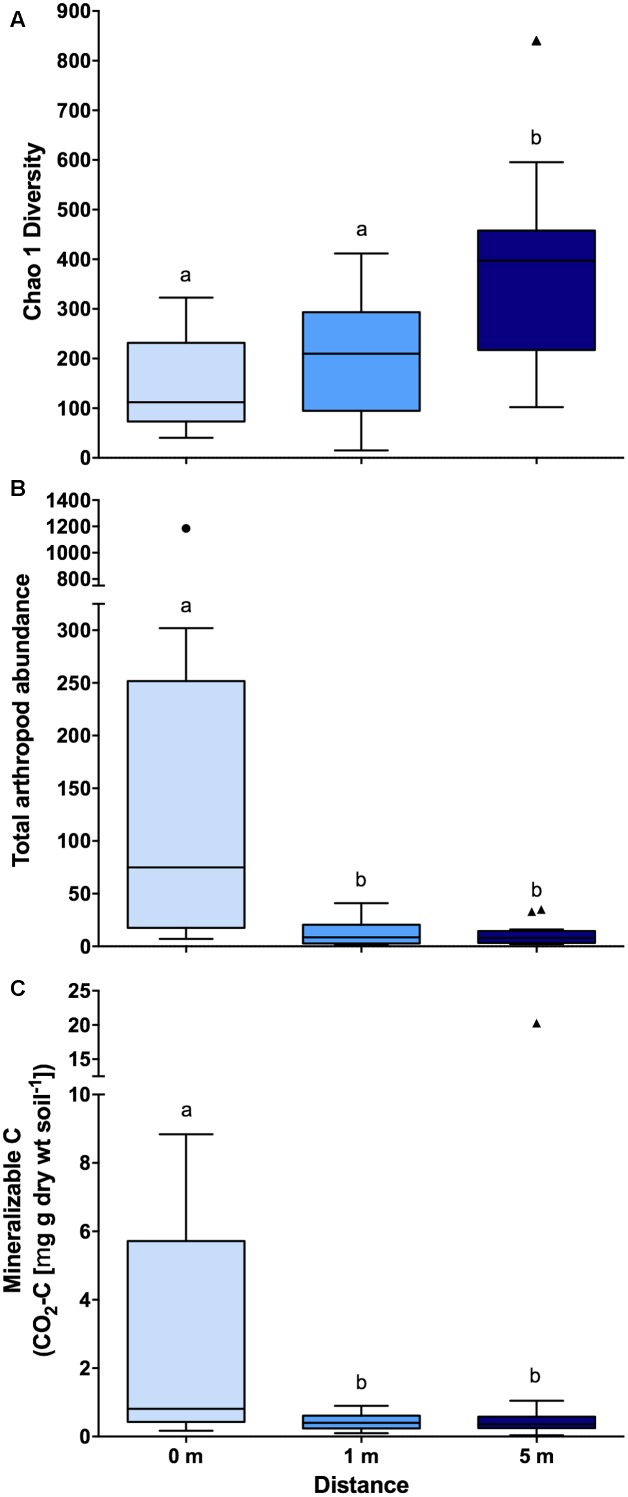
Boxplots showing **(A)** bacterial community diversity (*n* = 14 for 0 m distance and *n* = 17 for 1 and 5 m distances), **(B)** total arthropod (*n* = 19 for each distances) abundance (individuals 100 g soil^-1^), and **(C)** mineralizable-C (*n* = 19 for each distances) at each of the three sampling distances (i.e., 0, 1, and 5 m) from decomposing human cadavers. Outliers are denoted by symbols and letters indicate significant pairwise differences determined via Tukey HSD (*P* < 0.05). Also, note the break in the y-axis. Chao 1 diversity was calculated based on rarified OTU (97% identity threshold) table.

Across bacterial community composition, microbial function, and arthropod community composition we observed similar patterns at 0, 1, and 5 m distances from the cadaver, with the largest responses identified directly underneath the remains (**Figure 1**). The bacterial community composition at 0 m was significantly different (the significant effect of distance; *F*_2,45_ = 5.6; *P* < 0.001; **Figure 1A**), from both 1 and 5 m (*P* < 0.001 for both); whereas the 1 m community was not significantly different (*P* = 0.11) from the 5 m community. The differences between communities were driven by an increase in the relative abundance of classes Actinobacteria, γ-Proteobacteria, and Bacilli at 0 m samples compared to 1 and 5 m samples (**Figure 1B**). Together these three classes accounted for ∼30% of the dissimilarity between the 0 m samples and the 1 and 5 m samples. Similarly, in the presence of a cadaver (i.e., 0 m samples), the relative abundance of Bacteroidetes and Firmicutes was greater, while the relative abundance of Acidobacteria, Chloroflexi, Gemmatimonadetes, and Verrucomicrobia was lower when compared to the 1 and 5 m samples (**Supplementary Figure S2**) across time. Additionally, we found that the 0 and 1 m communities exhibited greater dispersion from the centroid than the 5 m community (*F*_2,45_ = 11.9; *P* < 0.001). This level of dispersion can be taken to indicate overall variability in multivariate datasets, but can also be taken as an indicator of β-diversity, meaning that communities at 0 and 1 m have greater β-diversity than those at 5 m. In contrast, we found that bacterial communities at 0 and 1 m had lower α-diversity when compared to communities at 5 m (*F*_2,35_ = 7.4; *P* < 0.01; **Figure 2A**).

Microbial community function was determined via CRP and a significant effect of distance from the cadaver (*F*_2,54_ = 3.21; *P* < 0.01; **Figure 1C**) was again found. The overall CRP was related to bacterial community composition (ρ = 0.36; *P* < 0.001). Function at 0 m differed from 1 to 5 m (*P* < 0.01 for both) and function at 1 and 5 m were not significantly different (*P* = 0.92). These differences were primarily driven by increased proportional respiration of citric acid and decreased proportional respiration of glucose at 1 and 5 m (**Figure 1D**). Together these differences in citric acid and glucose mineralization, accounted for ∼50% of the dissimilarity between the 0 m samples and the 1 and 5 m samples. We observed greater dispersion for community function at 0 m compared to 1 and 5 m (*F*_2,54_ = 12.08; *P* < 0.001).

Arthropod community composition also exhibited a significant effect with distance from the cadaver (*F*_2,33_ = 2.63; *P* < 0.001; **Figure 1E**), but overall, arthropod community composition was unrelated to either bacterial community composition (ρ = 0.13; *P* = 0.10) or microbial function (ρ = 0; *P* = 0.46). Arthropod community composition at 0 m differed from 1 to 5 m (*P* < 0.01 for both); however, community composition at 1 and 5 m did not differ significantly (*P* = 0.73) (**Supplementary Table S2**). These differences were primarily driven by increased relative abundance of the astigmatid mite family Acaridae (immature hypopi and adults) at 0 m compared to both 1 and 5 m (**Figure 1F**). Together acarids accounted for ∼33% of the dissimilarity between arthropod communities at 0 m and communities at the other two distances. Unlike both the bacterial community composition and function, no differences in dispersion were noted for the arthropod communities (*F*_2,33_ = 1.02; *P* = 0.47). With regards to absolute abundance, arthropods were significantly more abundant (15 to 17-fold) at the 0 m sampling distance compared to either the 1 or 5 m sampling distance (*F*_2,30_ = 15.8; *P* < 0.0001; **Figure 2B**).

In addition to bacterial and arthropod community composition, and microbial function, we also assessed soil pH, active microbial biomass, and mineralizable-C. We found no difference in either soil pH (*F*_2,44_ = 0.09; *P* = 0.91) or active microbial biomass (*F*_2,44_ = 1.06; *P* = 0.35) corresponding to the distance from the cadaver; however, for mineralizable-C, a significant effect of distance was observed (*F*_2,44_ = 8.15; *P* < 0.0001; **Figure 2C**). In fact, mineralizable-C, barring one outlier, was 6.8 and 7.4-fold greater at 0 m compared to 1 and 5 m, respectively.

### Temporal Dynamics

While strong spatial effects were associated with cadaver decomposition, we also investigated the temporal dynamics of decomposition on community composition and function directly under the cadaver (i.e., 0 m samples). Across the 732 days period of decomposition representing our study, we determined that cumulative precipitation (mm) compared to ADDs (in °C) was a more parsimonious (assessed via Akaike Information Criterion) determinant of decomposition dynamics and tended to explain a greater amount of variation in the dependent variables examined. Specifically, for the relationship between cumulative precipitation and citric acid respiration, mineralizable C, and the relative abundance of Actinobacteria, AIC was 8.13, 4.40, and 0.50 units lower, respectively, when compared to ADDs. This suggests that, at least for citric acid respiration and mineralizable C, there is considerably less support for a model including ADDs, as opposed to cumulative precipitation which was the best model given our data set (Burnham and Anderson, 2002). We also expected that examining the temporal dynamics of decomposition may help to explain the high level of dispersion associated with the 0 m samples.

**FIGURE 3:**
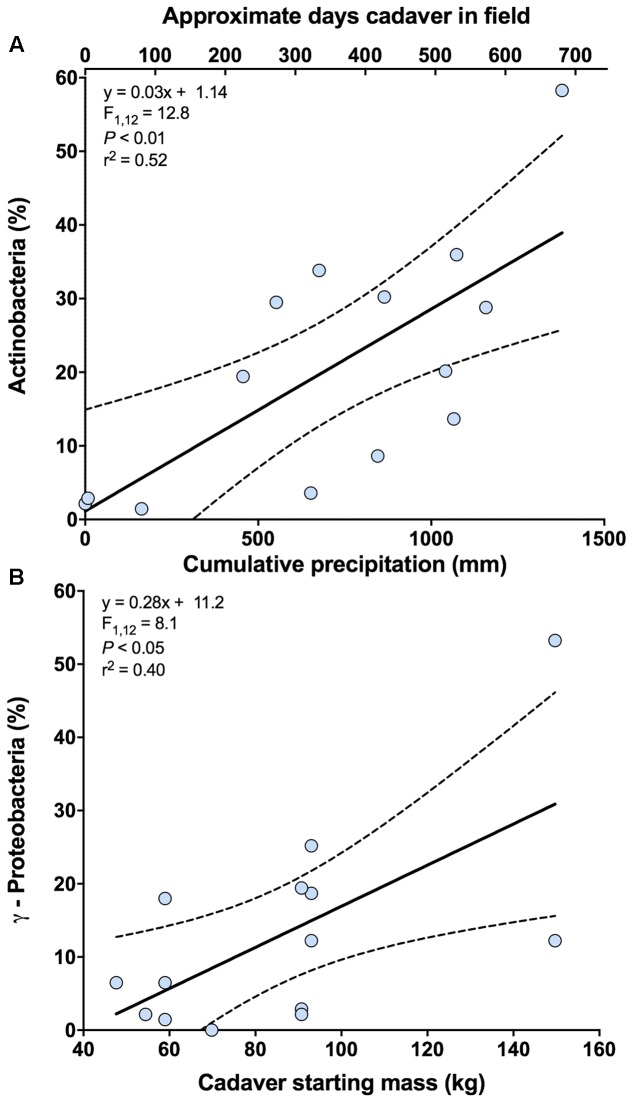
Relationships between bacterial classes and cadaver variables from 0 m samples. **(A)** Relationship between the relative abundance of class Actinobacteria and cumulative precipitation (mm) during the time course of cadaver decomposition. Also shown (upper x-axis) in this panel is the approximate number of days that a specific cadaver has been in the field. Note that approximate number of days is only an approximation and in some instances varied markedly from the cumulative precipitation associated with that cadaver. **(B)** Relationship between the relative abundance of class γ-Proteobacteria and cadaver starting mass. Dotted lines indicate 95% confidence intervals.

In the analysis of bacterial community composition, the first axis of the PCA was negatively related to cumulative precipitation (*y* = -0.03x - 12.26; *F*_1,12_ = 8.69; *P* < 0.05; *r*^2^ = 0.42). The second axis, which explains 6.9% of the variation in bacterial community composition, was unrelated to cumulative precipitation but a marginally significant relationship was noted between this axis and the starting weight of the cadaver (*y* = -0.14x + 25.60; *F*_1,12_ = 3.43; *P* = 0.09; *r*^2^ = 0.22). Further investigation of these results indicates that the class Actinobacteria is primarily related to the first principal coordinates axis, and classes γ-Proteobacteria and Bacilli are related to the second. For the relative abundance of Actinobacteria, a significant positive relationship with cumulative precipitation was observed (**Figure 3A** and **Supplementary Figure S1**). For γ-Proteobacteria, but not Bacilli (*F*_1,12_ = 3.22; *P* = 0.10), a significant positive relationship with cadaver starting mass (kg) was observed (**Figure 3B**).

**FIGURE 4:**
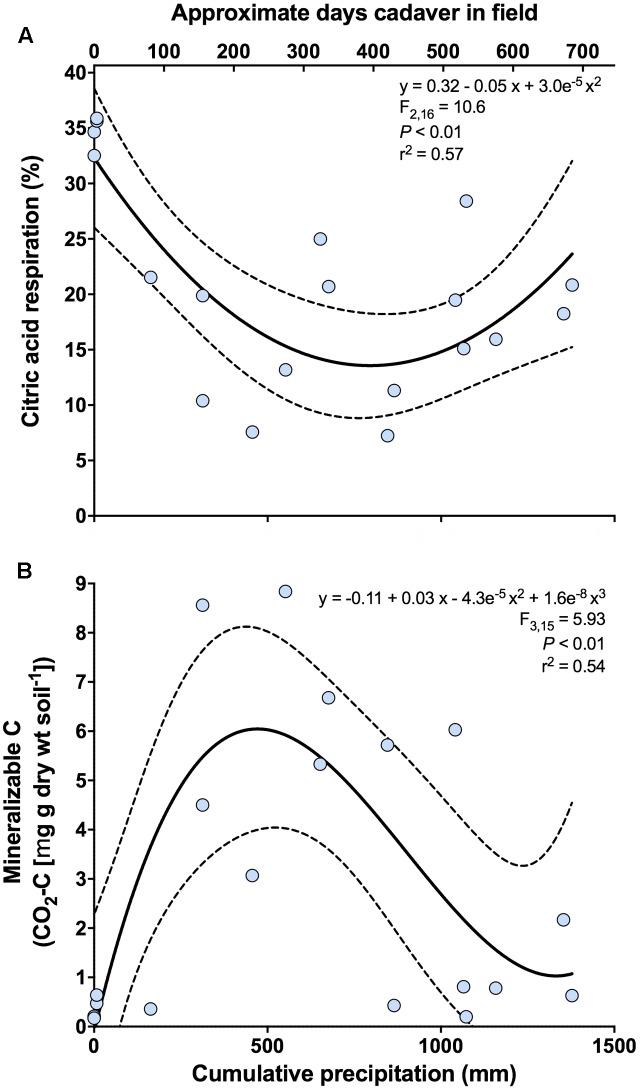
Change in the respiratory response of soil microbial communities at 0 m during the time-course of cadaver decomposition. **(A)** Relationship between proportional respiration of citric acid, determined via catabolic response profiling, and cumulative precipitation (mm) during the time course of cadaver decomposition from 0 m samples. Proportional mineralization of citric acid decreased during cadaver decomposition up until ∼600–700 (mm) of cumulative precipitation, at which point it began increasing again. **(B)** Relationship between mineralizable-C and cumulative precipitation (mm) during the time course of cadaver decomposition from 0 m samples. Mineralizable-C exhibited an increase up to ∼500 (mm) of cumulative precipitation, after which mineralizable-C decreased and subsequently appears to stabilize. Dotted lines indicate 95% confidence intervals. Also, shown (upper x-axis) is the approximate number of days that a specific cadaver has been in the field. Note that this is only an approximation and in some instances varied markedly from the cumulative precipitation associated with that cadaver.

For functional analyses, the first principal coordinates axis was significantly related to cumulative precipitation (*y* = -0.68 + 0.007x - 4.9e^-6^ x^2^; *F*_2,16_ = 6.90; *P* < 0.01; *r*^2^ = 0.46). No significant relationships were found for the second principal coordinates axis. Both the proportional mineralization of glucose and citric acid were related to the first axis. For glucose, we observed a marginally significant unimodal relationship with cumulative precipitation (*y* = 12.79 + 0.04x - 3.04e^-5^ x^2^; *F*_2,16_ = 2.93; *P* = 0.08; *r*^2^ = 0.27), where proportional glucose respiration peaked between 500 and 700 mm of cumulative precipitation (corresponding to ∼235–340 days since placement). For citric acid, we observed a significant bimodal relationship with cumulative precipitation (**Figure 4A** and **Supplementary Figure S1**), where proportional citric acid respiration peaked at 0 mm (∼3 days since placement) of cumulative precipitation and again at ∼1400 mm (∼700 days since placement).

Both bacterial community composition and function exhibited temporal relationships, whereas arthropod community composition did not. However, variation in soil pH, active microbial biomass, and mineralizable-C were all positively related to both principal coordinate axes (*P* < 0.05 and *r*^2^ ≥ 0.48 in all instances; **Supplementary Table S3**). The relative abundance of immature oribatid mites tended to be strongly correlated with both of these axes and exhibited the strongest relationship with active microbial biomass (*y* = 2.98x + 2.21; *F*_1,12_ = 10.26; *P* < 0.01; *r*^2^ = 0.51).

Both active microbial biomass (data not shown) and mineralizable-C were significantly related to cumulative precipitation. Both exhibited a unimodal response, with active microbial biomass peaking between 600 and 800 mm cumulative precipitation (corresponding to ∼290–390 days since placement) (*y* = -0.77 + 0.03x - 2.4e^-5^ x^2^; *F*_2,16_ = 4.43; *P* < 0.05; *r*^2^ = 0.36) (data not shown) whereas mineralizable-C peaked earlier at ∼500 mm cumulative precipitation (corresponding to ∼235 days since placement) (**Figure 4B**).

## Discussion

We demonstrated the introduction of decomposing vertebrate carrion (i.e., human) into an ecosystem has a profound impact on associated bacterial and arthropod community structure and microbial function. While these effects could be viewed as an expected response of the soil to the influx of large quantities of nutrients and associated microbes from the cadavers (Benninger et al., 2008; Metcalf et al., 2016), the spatial and temporal dynamics of such material on the soil ecosystem were not well-known. In fact, this is the first report of the impact of human cadaver decomposition on soil bacterial community structure and microbial function in conjunction with arthropod communities and associated soil characteristics: (1) beyond 198 days of decomposition, and (2) around the cadaver (1 and 5 m). Studies on non-human vertebrate carrion have measured similar responses, where carrion-associated changes in soil properties and plant communities are localized to the area below and directly adjacent decomposing carrion (Melis et al., 2007; Parmenter and MacMahon, 2009; Macdonald et al., 2014; Barton et al., 2016). Our results are also in agreement with other studies that show carrion decomposition can have a lasting effect on soil properties (Melis et al., 2007; Barton et al., 2016). The impact of cadavers across multiple soil communities and processes indicates the multi-trophic response to cadaver decomposition and a link between communities and function that can last for at least 15152 ADD (in °C) or 1378 cumulative precipitation (in mm) or 732 days. After the end of the experimental period (15152 ADD/1378 cumulative precipitation/732 days), C-mineralization and citric acid respiration almost returned to the background levels (**Figure 4**), while microbial community composition did not (**Figure 3A**). This disconnect between structure and function may indicate redundancy within the microbial community.

As previously observed (Metcalf et al., 2013, 2016; Cobaugh et al., 2015; Weiss et al., 2016), the soil bacterial community structure responded markedly to the presence of decomposing cadavers (**Figures 1A,B**, **2A**). Similar differences have been observed between rhizosphere and bulk soil (Kowalchuk et al., 2002), suggesting that increasing resource inputs may decrease bacterial diversity, especially when soil pH varies little across samples. Additionally, the observation that α-diversity was similar under and 1 m from the cadaver suggests the effect of decomposition is not limited to directly under the cadaver. In contrast, greater β-diversity associated with communities under the cadaver likely reflects variation associated with inter-cadaver antemortem microbial communities and differences associated with the decomposition progression (**Supplementary Table S1**). Such differences could markedly influence landscape scale variation in soil bacterial community composition during mass mortality events.

The response of the bacterial communities were similar to that detected from different soil types (Cobaugh et al., 2015). At the class level, Actinobacteria, γ-Proteobacteria, and Bacilli exhibited greater relative abundance under the cadaver. All three classes are known to be abundant in the human gut (Turnbaugh et al., 2009) and on necrophagous flies (Zheng et al., 2013; Singh et al., 2014), thus potentially explaining their greater relative abundance under the cadaver as opposed to samples at 1 and 5 m away from the cadaver (**Figures 1B,F** and **Supplementary Table S2**). Alternatively, these three classes are also known inhabitants of soils and may have become enriched given the significant changes in nutrient availability and soil chemistry induced by cadaver decomposition (Metcalf et al., 2016). Supporting this alternative, we observed a decrease in the relative abundance of bacterial classes within the phyla Acidobacteria and Verrucomicrobia, which are considered oligotrophic groups (Fierer et al., 2007, 2013). However, there is the likelihood that both mechanisms (i.e., introduction via the cadaver and insects and change in the soil environment) play a role in shaping bacterial community composition during cadaver decomposition (Crippen et al., 2015). For instance, during the course of cadaver decomposition, we observed an increase in the relative abundance of class Actinobacteria under the cadaver, which correlated with cumulative precipitation (**Figure 3A**). This enrichment suggests that this bacterial class may be selected for over the decomposition process, but its abundance is also dependent on environmental conditions, in this case, precipitation. On the other hand, the relative abundance of class γ-Proteobacteria in the soil was higher with a larger initial biomass of the cadaver (**Figure 3B**). This conflicts with Weiss et al. (2016), who didn’t find a significant relationship between cadaver mass and grave soil bacterial community structure. Differences in experimental design between this study and that used by Weiss et al. (2016), make it difficult to compare findings of two studies: they used porcine cadavers (body mass ranged from 1 to 50 Kg) for a total 15 days of decomposition period, whereas we used human cadavers (body mass ranged from 47 to 153 Kg) for a total 732 days/1378 ADD (°C) of decomposition period. Lecomte et al. (2015) observed significantly high relative abundance of γ-Proteobacteria in high fat diet (HFD) fed rats (had significantly higher weight) compared to control chow diet fed rats. Although, in terms of size and body mass, rats and humans are not comparable, this study does support the idea that the γ-Proteobacteria may be introduced to the soil via the cadaver (i.e., the greater the cadaver mass the more γ-Proteobacteria introduced when the cadaver purges into the soil). Alternatively, this may suggest underlying edaphic factors selecting for γ-Proteobacteria could be altered by cadaver mass or composition, although we observed no evidence for this in the measurements we conducted.

Microbial community function, as assessed via CRP, was also strongly affected by cadaver decomposition (**Figures 1C,D**). As with bacterial community composition, this increase in dispersion is likely to lead to greater heterogeneity in function across a landscape experiencing cadaver inputs. Differences between distances were affected by greater relative respiration of glucose, and lower citric acid respiration, under the cadaver compared to either 1 or 5 m from the cadaver. This difference in relative respiration may be driven by the more copiotrophic communities observed under the cadavers and greater availability of mineralizable-C (**Figure 2C**), which explains the increased utilization of glucose. Citric acid may have a lower energy yield but is likely in greater abundance in soils under normal conditions (van Hees et al., 2005), explaining its greater utilization at 1 and 5 m from the cadaver (**Figure 4A**).

Changes in both function and bacterial community composition are partly driven by increased resource availability (**Figure 2C**). As others have shown, decomposing cadavers represent a major source of C (and nutrients) to soil microbial communities (Yang, 2004); but we find that this resource pulse is both dynamic and relatively long lasting (**Figure 4B**). Surprisingly, the increase in mineralizable-C under the cadaver did not coincide with a similar increase in active microbial biomass. One potential explanation for this lack of an increase in microbial biomass, is that microbial biomass was suppressed via either top–down regulation (i.e., microbivory) or resource competition. We observed significantly greater abundance of microbivorous arthropods (e.g., Isotomidae, Uropodidae, etc.) under cadavers compared to 1 and 5 m away (**Supplementary Table S2**), and a concomitant increase in the relative abundance of astigmatid mites, which commonly feed on bacteria and fungi (Philips, 1990) (**Figures 1E,F**). Our sampling design was insufficient to dissect the details of these potential interactions further, highlighting an area where this type of research could be extended.

One of the compelling results from this study was the observation (based on AIC values of the models tested) that the starting mass of a cadaver and associated precipitation (**Figures 3A,B**) can, in some instances, explain bacterial taxon abundance more effectively than ADDs. There are several practical consequences of these observations related to understanding, management, and interpretation of soil bacterial communities. First, it is clear that different taxa appear sensitive to different variables, such that accumulated heat, accumulated precipitation, and carrion biomass (at the size scales studied here) should all be considered in future studies. However, it is also clear that not all members of the community are equally sensitive to an abiotic pressure (e.g., precipitation). In addition, the taxa in this study sensitive to cumulative precipitation are the most likely candidate taxa to impact precipitation dependent soil functions like citric acid respiration and mineralizable-C production. Another consequence of these observations stems from the fact that different ecoregions of the world have different distributions of potential carcass size and frequency of inputs. For instance, some ecosystems have more megafauna or experience more regular mass die offs than others (Beasley et al., 2015; Jones et al., 2015), which the results of this study would predict should result in different soil bacterial communities and functions than others. Unlike most systems, some areas received predictable resource pulses (Yang et al., 2010), which could select for microbial communities responsive to such resources. This concept has been well-studied for cicadas (Yang, 2004, 2006, 2009, 2013), salmon runs (Gende et al., 2002; Moore et al., 2007; Pechal and Benbow, 2016), and even the great wildebeest migration in Africa (Jones et al., 2015; Subalusky et al., 2017); however, the impact of such predictable resources on the microbial community is truly underappreciated at this time.

The results of this study begin to shed light on the specific responses of soil communities that respond to carrion inputs across applications derived from microbial communities. For instance, in forensic science there is a growing body of evidence that bacterial communities may be useful in determining how long someone has been deceased (Metcalf et al., 2013; Pechal et al., 2013; Iancu et al., 2015). However, models predicting how long remains have been decomposing with microbial community data are typically implemented using ADD. Our results suggest predictive models of a postmortem interval with such data may be more accurate or more precise if they include accumulated precipitation and the starting weight of a decedent. Accordingly, this study has identified empirical support for key features that are likely necessary for understanding, managing, and interpreting soil bacterial community information associated with decomposing remains.

These data represent the first integration of multiple trophic levels associated with decomposing human remains over an extended period of time (732 days) into a single analysis. We determined that human cadaver decomposition alters soil bacterial communities structure and microbial function directly under cadavers, and that this effect diminishes with increasing distance from the cadaver. Such information is critical to understand ecosystem function considering vertebrate carrion are a natural part of most ecosystems and the increased number of mass mortality events occurring globally (Fey et al., 2015). Future studies will need to extend such investigations to greater temporal scales and frequency of sampling to determine if soil systems recover or shift to a unique stable pattern. The consequences of these discoveries could have a profound influence on the approaches taken for examining ecosystem sensitivity and resilience, as well as the response of soil communities and the ecosystem processes to cadaver inputs.

## Author Contributions

BS, KM, MS, KW, TC, AT, MB, JT, and JP designed the study. BS, TC, AT, JT, and JP collected soil samples for all analyses. BS and TC performed DNA extraction for 16S rDNA 454 sequencing. BS, TC, NS, MS, and AT performed sequence data analysis for the determination of bacterial structure. KM and MS performed data analysis for the determination of microbial function. KW performed arthropod extraction and data analysis. All authors contributed, reviewed, and approved the manuscript.

## Conflict of Interest Statement

The authors declare that the research was conducted in the absence of any commercial or financial relationships that could be construed as a potential conflict of interest.

## Supplementary material

FIGURE S1 Relationships between the number of days since the cadaver was placed in the field (age) and **(A)** accumulated degree days (in °C); and **(B)** cumulative precipitation (in mm). Note the slightly greater variation associated with cumulative precipitation, which likely explain its more parsimonious relationship with variables determined during the time course of cadaver decomposition.

FIGURE S2 Average compositional differences at the phyla level bacterial communities at each measurement distance from cadavers (*n* = 14 for 0 m distance and *n* = 17 for 1 and 5 m distances).

TABLE S1 List of cadavers showing death information and soil properties associated with each cadaver and sample collection date.

TABLE S2 Soil arthropod densities (individuals 100 g^-1^ soil ± SE) under (0 m), and at 1 (1 m) and 5 (5 m) distances from cadavers.

TABLE S3 Linear regression results for the first two axes scores of the arthropod principal coordinates analysis and active microbial biomass, soil pH, and mineralizable-C.
