# Supplementary figures and images for: Temporal and Spatial Impact of Human Cadaver Decomposition on Soil Bacterial and Arthropod Community Structure and Function
Source: Front Microbiol. 2018 Jan 4;8:2616. doi: 10.3389/fmicb.2017.02616 (PMC5758501; doi:10.3389/fmicb.2017.02616)

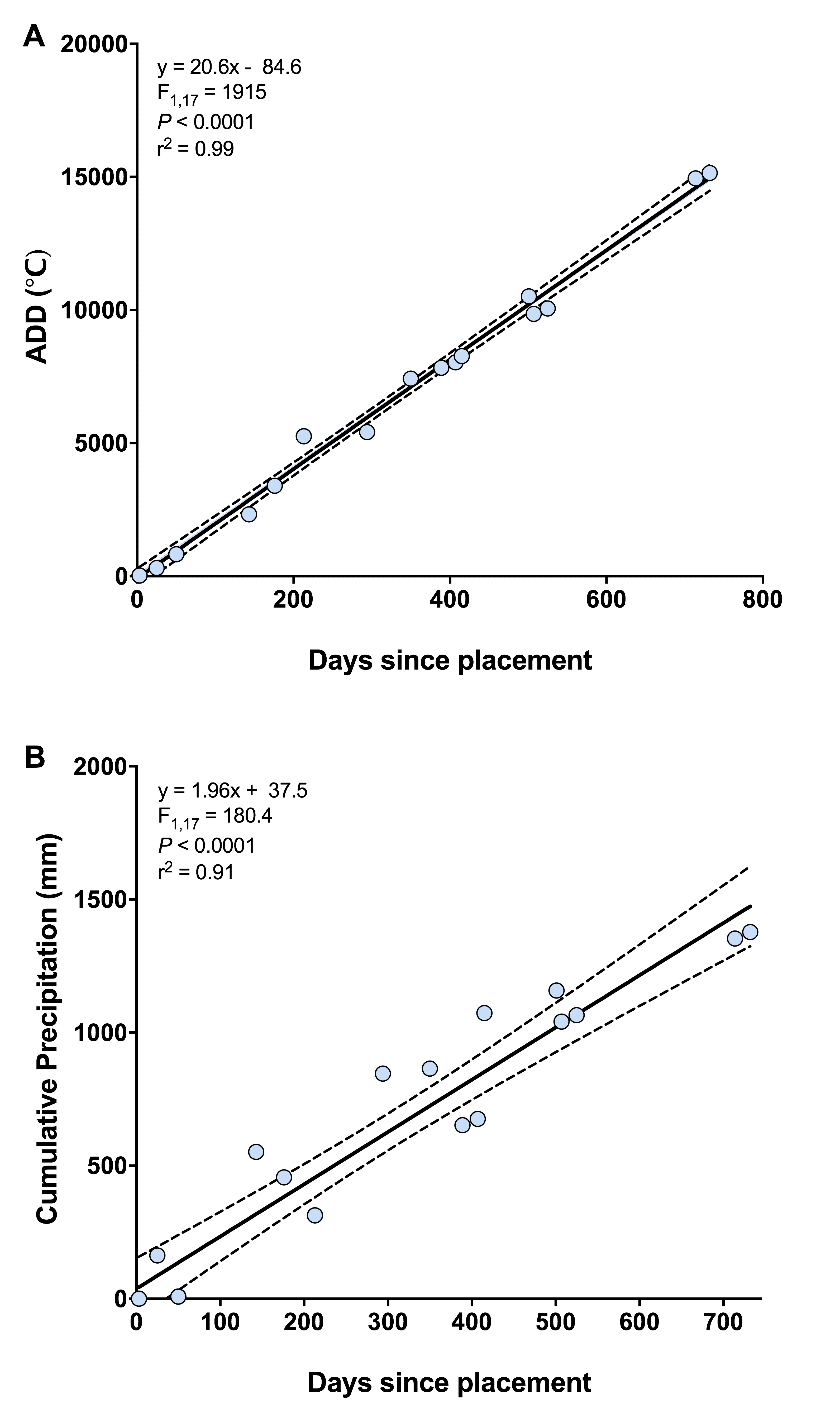

Supplement: FIGURE S1 — Relationships between the number of days since the cadaver was placed in the field (age) and (A) accumulated degree days (in °C); and (B) cumulative precipitation (in mm). Note the slightly greater variation associated with cumulative precipitation, which likely explain its more parsimonious relationship with variables determined during the time course of cadaver decomposition. [file Image_1.TIFF]

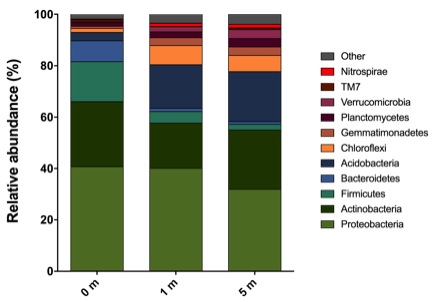

Supplement: FIGURE S2 — Average compositional differences at the phyla level bacterial communities at each measurement distance from cadavers (n = 14 for 0 m distance and n = 17 for 1 and 5 m distances). [file Image_2.JPEG]
