# Supplementary material for: Temporal and Spatial Impact of Human Cadaver Decomposition on Soil Bacterial and Arthropod Community Structure and Function
Source: Front Microbiol. 2018 Jan 4;8:2616. doi: 10.3389/fmicb.2017.02616 (PMC5758501; doi:10.3389/fmicb.2017.02616)
Supplement: TABLE S2 — Soil arthropod densities (individuals 100 g-1 soil ± SE) under (0 m), and at 1 (1 m) and 5 (5 m) distances from cadavers. [file Table_2.DOCX]

**Table S2.**  Soil arthropod densities (individuals 100 g^-1^ soil ± SE) under (0 m), and at 1 (1 m) and 5 meter (5 m) distances from cadavers. Data were analyzed using mixed model analysis of variance with location and sample date as fixed factors and donor as a random factor. Post hoc analyses among locations (0, 1, and 5m) were made using LS means comparisons.

| taxa | 0 m | 1 m | 5 m | F | *P* |
| --- | --- | --- | --- | --- | --- |
| Formicidae | 0.0 (0.0)b | 0.0 (0.0)b | 0.3 (0.2)a | 8.1 | 0.01 |
| Carabidae (juv) | 1.6 (0.6)a | 0.2 (0.1)b | 0.0 (0.0)b | 21.6 | 0.0004 |
| Diptera (juv) | 1.3 (0.6)a | 0.0 (0.0)b | 0.0 (0.0)b | 9 | 0.007 |
| Isotomidae | 6.3 (4.2)a | 0.3 (0.1)b | 0.6 (0.4)b | 4.5 | 0.04 |
| Mesostigmata (pred) | 6.3 (2.6)a | 1.1 (0.5)b | 0.7 (0.3)b | 8.9 | 0.007 |
| Uropodidae | 6.2 (3.5)a | 0.0 (0.0)b | 0.0 (0.0)b | 5.6 | 0.03 |
| Acaridae | 47.4 (18.8)a | 1.9 (0.9)b | 1.5 (0.4)b | 19.8 | 0.001 |
| Acaridae (hypopi) | 120.3 (86.2)a | 0.8 (0.4)b | 0.8 (0.4)b | 4.5 | 0.05 |
| Oribatida | 1.0 (0.4) | 6.3 (2.3) | 6.2 (1.7) | 3.03 | 0.09 |
| -Opiidae | 0.6 (0.4) | 1.3 (0.6) | 2.4 (1.2) | 2.23 | 0.16 |
| -Phthiricaridae | 0.0 (0.0) | 0.7 (0.3) | 0.1 (0.1) | 2.33 | 0.15 |
| Prostigmata | 1.6 (1.0) | 1.6 (0.9) | 0.7 (0.5) | 0.66 | 0.54 |
| Staphylinidae | 0.5 (0.4) | 0.2 (0.2) | 0.0 (0.0) | 1.21 | 0.34 |
| Total Abundance | 194.5 (94.6)a | 12.8 (3.6)b | 11.4 (3.3)b | 8.4 | 0.009 |
| ^letters denote significant differences based on post hoc comparisons^ | | | | |  |
| ^juv – juvenile, pred - predatory^ |  |  |  |  |  |
