# Supplementary material for: Temporal and Spatial Impact of Human Cadaver Decomposition on Soil Bacterial and Arthropod Community Structure and Function
Source: Front Microbiol. 2018 Jan 4;8:2616. doi: 10.3389/fmicb.2017.02616 (PMC5758501; doi:10.3389/fmicb.2017.02616)
Supplement: TABLE S3 — Linear regression results for the first two axes scores of the arthropod principal coordinates analysis and active microbial biomass, soil pH, and mineralizable-C. [file Table_3.DOCX]

**Table S3.** Linear regression results for the first two axes scores of the arthropod principal coordinates analysis and active microbial biomass, soil pH, and mineralizable-C.

| **Variable 1** | **Variable 2** | **Equation** | **F_1,10_** | **r^2^** | ***P*-value** |
| --- | --- | --- | --- | --- | --- |
| Axis 1 | Microbial biomass | Y = 0.23x + 5.7 | 20.5 | 0.67 | **<0.01** |
|  |  |  |  |  |  |
|  | Soil pH | Y = 0.02x + 6.5 | 9.2 | 0.48 | **<0.05** |
|  |  |  |  |  |  |
|  | Mineralizable-C | Y = 0.08x + 1.9 | 19.2 | 0.66 | **<0.01** |
|  |  |  |  |  |  |
| Axis 2 | Microbial biomass | Y = 0.37x + 15.3 | 19.3 | 0.66 | **<0.01** |
|  |  |  |  |  |  |
|  | Soil pH | Y = 0.04x + 7.6 | 10.7 | 0.52 | **<0.01** |
|  |  |  |  |  |  |
|  | Mineralizable-C | Y = 0.13x + 5.4 | 15.7 | 0.61 | **<0.01** |
|  |  |  |  |  |  |
